# YTHDF1 promotes hepatocellular carcinoma progression via activating PI3K/AKT/mTOR signaling pathway and inducing epithelial-mesenchymal transition

**DOI:** 10.1186/s40164-021-00227-0

**Published:** 2021-06-04

**Authors:** Xiangyuan Luo, Mengdie Cao, Fan Gao, Xingxing He

**Affiliations:** 1 Institute of Liver and Gastrointestinal Diseases, Tongji Hospital, Tongji Medical College, Huazhong University of Science and Technology, Wuhan, 430030 China; 2 Hubei Key Laboratory of Hepato-Pancreato-Biliary Diseases, Tongji Hospital, Tongji Medical College, Huazhong University of Science and Technology, Wuhan, 430030 China

**Keywords:** Liver cancer, YTHDF1, m^6^A, PI3K/AKT/mTOR, EMT

## Abstract

**Background:**

*N*^6^-methyladenosine (m^6^A) modification, as the most abundant RNA modification, widely participates in the physiological process and is involved in multiple disease progression, especially cancer. YTH N6-methyladenosine RNA binding protein 1 (YTHDF1) is a pivotal m^6^A “reader” protein, which has been reported in multiple cancers. However, the role and molecular mechanism of YTHDF1 in HCC are still not fully elucidated.

**Methods:**

Based on various bioinformatics databases, q-RT PCR, western blot, and a tissue microarray containing 90 HCC samples, we examined the expression of YTHDF1 in HCC. Then, we applied the loss-of-function experiments to explore the role of YTHDF1 in HCC by in vitro and in vivo assays. Finally, we performed the gene set enrichment analysis (GSEA) to predict the potential signaling pathway of YTHDF1 involved in HCC and further verified this prediction.

**Results:**

YTHDF1 was overexpressed in HCC and associated with HCC grade. Depletion of YTHDF1 markedly impaired the proliferation, migration, invasion, and cell cycle process of HCC cells. Mechanistically, YTHDF1 promoted the growth of HCC cells via activating the PI3K/AKT/mTOR signaling pathway. Moreover, we also demonstrated that the epithelial-mesenchymal transition (EMT) mediated the promoting effect of YTHDF1 on the migration and invasion of HCC cells.

**Conclusions:**

YTHDF1 contributes to the progression of HCC by activating PI3K/AKT/mTOR signaling pathway and inducing EMT.

**Supplementary Information:**

The online version contains supplementary material available at 10.1186/s40164-021-00227-0.

## Background

Hepatocellular carcinoma (HCC) is the sixth most common cancer and the fourth leading cause of cancer-related mortality globally, which has caused a severe health burden globally [1, 2]. Although the development of novel drugs for HCC has taken a historic step in light of the approval of several multi-kinase inhibitors and immune inhibitors in recent years, the mortality of patients with HCC remains highly [3–5].

It has been well verified that epigenetic modification is significant in bioprocesses and disease progression. Among the over 100 kinds of chemical modifications found in various RNAs, N6-methyladenosine (m^6^A) is the most abundant internal RNA modification [6, 7]. m^6^A modification is dynamically recognized and regulated by “writer”, “eraser”, and “reader” proteins [8]. “Writers” represent multiple methyltransferases, including the essential components methyltransferase like 3 and 14 (METTL3/METTL14) and other subunits WT1 associated protein (WTAP) and KIAA1429 [6, 8, 9]. As a reversible modification, m^6^A is removed by demethylases (“erasers”) belonging to the AlkB family proteins of dioxygenases, specifically the FTO alpha-ketoglutarate dependent dioxygenase (FTO) and AlkB homolog 5, RNA demethylase (ALKBH5) [6, 8]. The “readers” are mainly responsible for recognizing and binding to m^6^A-modified RNAs and affecting their fate and function through post-transcriptional regulation.

YTH N6-methyladenosine RNA binding protein 1 (YTHDF1), as a pivotal m^6^A “reader,” mainly acts as a translation initiation promotor by interacting with initiation factor eIF3 [10]. As the role of YTHDF1 in m^6^A modification has been revealed, more studies have described the effects of YTHDF1 on disease progression, especially cancer. Specifically, studies have shown that YTHDF1 is high-expressed in multiple malignancies and promotes the progression of colorectal carcinoma, ovarian cancer, gastric cancer, non-small cell lung cancer, and hepatocellular carcinoma through a range of oncogenic mechanisms [11–16]. Notably, it has also been reported that the loss of YTHDF1 strengthens the cross-presentation of tumor antigens and the cross-priming of CD8 + T cells, and enhances the blocking effect of the programmed cell death 1 ligand 1 (PD-L1) checkpoint in vivo, suggesting that YTHDF1 functions as a potential therapeutic target [17]. However, the role and molecular mechanism of YTHDF1 in HCC are still not fully elucidated.

In this study, we identified that YTHDF1 was significantly overexpressed and associated with a poor prognosis in HCC by bioinformatics analysis and tissue microarrays, and further revealed that YTHDF1 activated the PI3K/AKT/mTOR signaling pathway and induced EMT, leading to the enhanced proliferation, migration and invasion of HCC cells.

## Methods

### Tissue specimens and cell culture

After obtaining informed consent and complying with ethical and institutional guidelines, all of 12 tumorous and matched adjacent nontumorous liver tissue specimens were collected from patients with HCC who underwent liver resection due to liver cancer at the Tongji Hospital, Huazhong University of Science and Technology. All cell lines were cultured in DMEM supplemented with 10% fetal bovine serum (Cegrogen, Germany) in a humidified atmosphere with 5% CO_2_ at 37 °C.

### Cell transfection, cell treatment, and chemical reagents

si-YTHDF1s and negative control siRNA-NC were acquired from Guangzhou Ribobio, China. The selected sequences of siRNAs were as follows: si-YTHDF1-1: 5′-GGAACAACATCTATCAGCA-3′, si-YTHDF1-2: 5′-GCTCAACCGCAGTATCAGA-3′, si-YTHDF1-3: 5′-GGAAACGTCCAGCCTAATT-3′. The establishment of sh-YTHDF1 cell line was co-transfect the vector pLKO.1 expressing sh-YTHDF1 with the packaging vectors psPAX and vesicular stomatitis virus-expressing envelope vector pMD2G in HEK293T cells for 72 h to produce lentiviral particles. After incubating lentivirus in tumor cells for 48 h with polybrene, 1 µg/ml puromycin (Biosharp, China) was used to screen stable cell lines. Cells treated with Human TGF-beta1 (PeproTech, 100-21-10) at 10 ng/ml for 48 h or treated with SC79 (MCE, HY-18749) at 4 µg/ml for 1 h. Cell transfections were conducted using Lipofectamine 3000 (Invitrogen, USA) according to the instructions and protocols.

### Tissue microarrays and immunofluorescence assay

A hepatocellular carcinoma tissue microarray containing 90 tumorous tissues and adjacent nontumorous tissues was acquired from the Shanghai Outdo Biotech (HLiv-H180Su08). The immunohistochemical (IHC) staining of YTHDF1 in these 90 cases was performed as described previously [18]. The IHC staining score (the multiplication of staining intensity and positive percentage) was used to evaluate the expression of YTHDF1. The staining intensity: negative = 0, weak = 1, moderate = 2, and strong = 3; The positive percentage: negative = 0, 1–25% = 1, 26–50% = 2, 51–75% = 3, and 76–100% = 4. IHC staining scores ≥ 4 defined high expression, while scores < 4 defined low expression. The anti-YTHDF1 antibody used in IHC was purchased from Proteintech (17479-1-AP).

The subcellular localization of YTHDF1 was performed by immunofluorescence assay as previously described [19]. Briefly, the cells cultured on the coverslips were washed three times using PBS, then fixed with 4% paraformaldehyde for 20 min, and permeabilized cells by 0.3% Triton X-100 for 10 min. After washing three times with PBS, the cells were blocked in 10% goat serum for 40 min and incubated with anti-YTHDF1 (Proteintech, China) at 4 °C overnight. Cells were washed with PBS for three times and incubated with the secondary antibodies (Proteintech, China) for 2 h. Cell nuclei were stained with DAPI for 5 min. Finally, the fluorescence images were observed by fluorescence microscope.

### Western blot assay

The protein was dissociated in RIPA buffer (Promoter, Wuhan, China) containing PMSF (Promoter, Wuhan, China) and protease inhibitors cocktail (MCE, USA) for 30 min on ice. The cell lysates were centrifuged at 4 °C for 10 min at high speed. After removing the precipitate, the supernatant was collected to measure protein concentration by BCA kit (Servicebio, Wuhan, China). After boiling the protein added with SDS loading buffer, the protein samples were electrophoresed and transferred onto PVDF membranes. The membranes were blocked by 5% non-fat milk dissolved in TBST for 1.5 h and were then incubated with primary antibodies at 4 °C overnight. After washing the membranes by TBST three times, we incubated them with secondary antibodies for 1.5 h. The images were developed by an imaging system (Tanon 5200 Multi, China). The antibodies used in this study were as follows: anti-YTHDF1 (Proteintech, 17479-1-AP), anti-MMP2 (Proteintech, 10373-2-AP), anti-MMP9 (CST, 13667), anti-Claudin 1 (Bioswamp, PAB33267), anti-ZO-1 (Bioswamp, MAB43858), anti-N-cadherin (Bioswamp, PAB30130), anti-Vimentin (CST, 3390), anti-AKT (CST, 4691), anti-phospho-AKT (CST, 4060), anti-AKT1 (Proteintech, 51077-1-AP), anti-AKT2 (Proteintech, 17609-1-AP), anti-AKT3 (Proteintech, 21641-1-AP), anti-mTOR (CST, 2983), anti-phospho-mTOR (CST, 5536), anti-tubulin (CST, 2125), anti-Cyclin D1 (Proteintech, 60186-1-Ig), anti-CD44 (CST, 37259), anti-GAPDH (Proteintech, 60004-1-lg), anti-beta Actin (Proteintech, 20536-1-AP).

### RNA isolation and quantitative real-time PCR (q-RT PCR)

Total RNA was isolated by trizol reagent (Invitrogen, Carlsbad, America). The reverse transcription of RNA into cDNA was performed using HiScript^®^ II Q Select RT SuperMix for qPCR (Vazyme, Nanjing, China). QuantStudio 3 Real-Time PCR system (Applied Biosystems, USA) was used for q-RT PCR to detect RNA expression by Hieff^®^ qPCR SYBR Green Master Mix (Yeasen, Shanghai, China). The q-RT PCR primers were as follows: YTHDF1 forward 5′-ATACCTCACCACCTACGGACA-3′, YTHDF1 reverse 5′-GTGCTGATAGATGTTGTTCCCC-3′, AKT1 forward 5′-GCCCAACACCTTCATCATCC-3′, AKT1 reverse 5′-ACTCCTCCCGCTCCTCAG-3′, AKT2 forward 5′-GCGGAAGGAAGTCATCATTG-3′, AKT2 reverse 5′-GTGGGTCTGGAAGGCATAC-3′, AKT3 forward 5′-TTCTCTGGAGTAAACTGGCAAG-3′, AKT3 reverse 5′-TGGTGTTATTGTAATAGTCTGAGC-3′, ACTIN forward 5′-CATGTACGTTGCTATCCAGGC-3′, ACTIN reverse 5′-CTCCTTAATGTCACGCACGAT-3′.

### Cell proliferation assay

Cell proliferation ability was measured by CCK8 assay, colony-forming assay, and EdU staining assay. The CCK8 assay was to culture 1000 cells per well into 96-well plates overnight. The cell culture medium and CCK-8 (Promoter, Wuhan, China) were mixed with a 10:1 ratio to incubate the cells at 37 °C for 1 h, 2 h, 3 h, and 4 h, and then measured the 450 nm absorbance by a microplate reader. These steps were repeated at 24 h, 48 h and 72 h. As to colony-forming assay, 1000 cells per well were cultured in 6-well plate for 14 days. After washing the plate 3 times with PBS, we used paraformaldehyde to fix for 10 min and 0.5% crystal violet to stain cells for 10 min. EdU staining assay was performed by 5-ethynyl-2′-deoxyuridine (EdU) assay kit (Ribobio, Guangzhou, China). First, 1 × 10^5^ cells per well were cultured in a 96-well plate and incubated with 50 μM EdU buffer at 37 °C for 2 h. After washing the plate with PBS, the cells were fixed with 4% paraformaldehyde and permeabilized with 0.5% Triton X-100. Then the cells were incubated by Apollo stain mixture for 30 min and washed with 0.5% Triton X-100 3 times. The nuclei were stained with Hoechst 33342 reaction solution for 30 min. The images were observed by fluorescence microscope.

### Cell cycle assay

5 × 10^5^ cells per well were cultured in a 6-well plate. After digesting and collecting cells, these cells were fixed with 75% ice ethanol at − 20 °C overnight. After centrifuging cells to remove ethanol, the cells were washed with ice PBS. Then these cells were incubated with a 400 µl mixture of propidium iodide (PI) and RNase A (KeyGen Biotech, Nanjing, China) at room temperature over 30 min. The results were analyzed by a FACSAria cell sorting system (BD Biosciences, San Jose, CA, USA).

### Cell migration and invasion assay

Transwell assay and wound healing assay were used to measure the cell migration and invasion. In transwell assay, 4 × 10^4^ cells per well were seeded in a 24-well plate equipped with 8-μm pore size transwell inserts (Corning, NY, USA) with or without Matrigel (Corning, 354234). The upper chamber was filled in a serum-free medium with cells, and the bottom chamber was filled with a serum-contain medium without cells. After incubating for 24 h, the medium was removed and the membrane of inserts was washed with PBS. The cells on the membrane were fixed with paraformaldehyde and stained with 0.5% crystal violet. The images were observed by a light microscope. For wound healing assay, 1 × 10^6^ cells were seed to each well in a 12-well plate. Then, the cells in each well were scratched by plastic tips and the images were captured by light microscope. After incubating 24 h, the cell movement was captured by light microscope.

### Bioinformatics analysis

Bioinformatics websites include GEPIA (http://gepia.cancer-pku.cn), UALCAN (http://ualcan.path.uab.edu/index.html), LinkedOmics (http://www.linkedomics.org/admin.php), and Kaplan–Meier Plotter (http://kmplot.com/analysis/index.php?p=service&cancer=liver_rnaseq).

We downloaded the samples of patient with HCC from The Cancer Genome Atlas (TCGA, https://www.cancer.gov/tcga/). The sample included 186 cases of high YTHDF1 expression and 185 cases of low YTHDF1 expression. These sample data were processed by Gene Set Enrichment Analysis software (GSEA v4.0.1) to implement BIOCARTA analysis, HALLMARK analysis, and Kyoto Encyclopedia of Genes and Genomes (KEGG) analysis as previously described [20].

### Subcutaneous implantation experiment

All animal experiments were approved by the Animal Ethics Committee of the Tongji Hospital of Huazhong University of Science and Technology. Ten four-week-old BALB/c male nude mice (GemPharmatech, Jiangsu, China) were subcutaneously injected with control Huh7 cells 2 × 10^6^ (left-back) and stable knockdown of YTHDF1 Huh7 cells 2 × 10^6^ (right-back). These cells were respectively premixed with 50 µl Matrigel (Corning, 354,234) in 100 µl PBS. Then the tumor size was measured every 5–8 days. When the tumors have grown to an appropriate size (about 30 days), they were taken out for weight measurement and volume estimation (volume = 1/2 × length × width^2^).

### Statistical analysis

All experiments were presented as means ± SD from independently repeated experiments at least three times. The experimental data were analyzed by GraphPad Prism 7.0 and SPSS 16.0. The difference between two groups was compared using a 2-tailed Student’s *t*-test, the survival analysis was evaluated by log-rank test, the correlation analysis was measured by Pearson correlation test. The *P*-value < 0.05 indicates that the difference is statistically significant (*P < 0.05; **P < 0.01; ***P < 0.001; ****P < 0.0001).

## Results

### YTHDF1 was high-expressed in HCC and correlated with poor survival

**Fig. 1:**
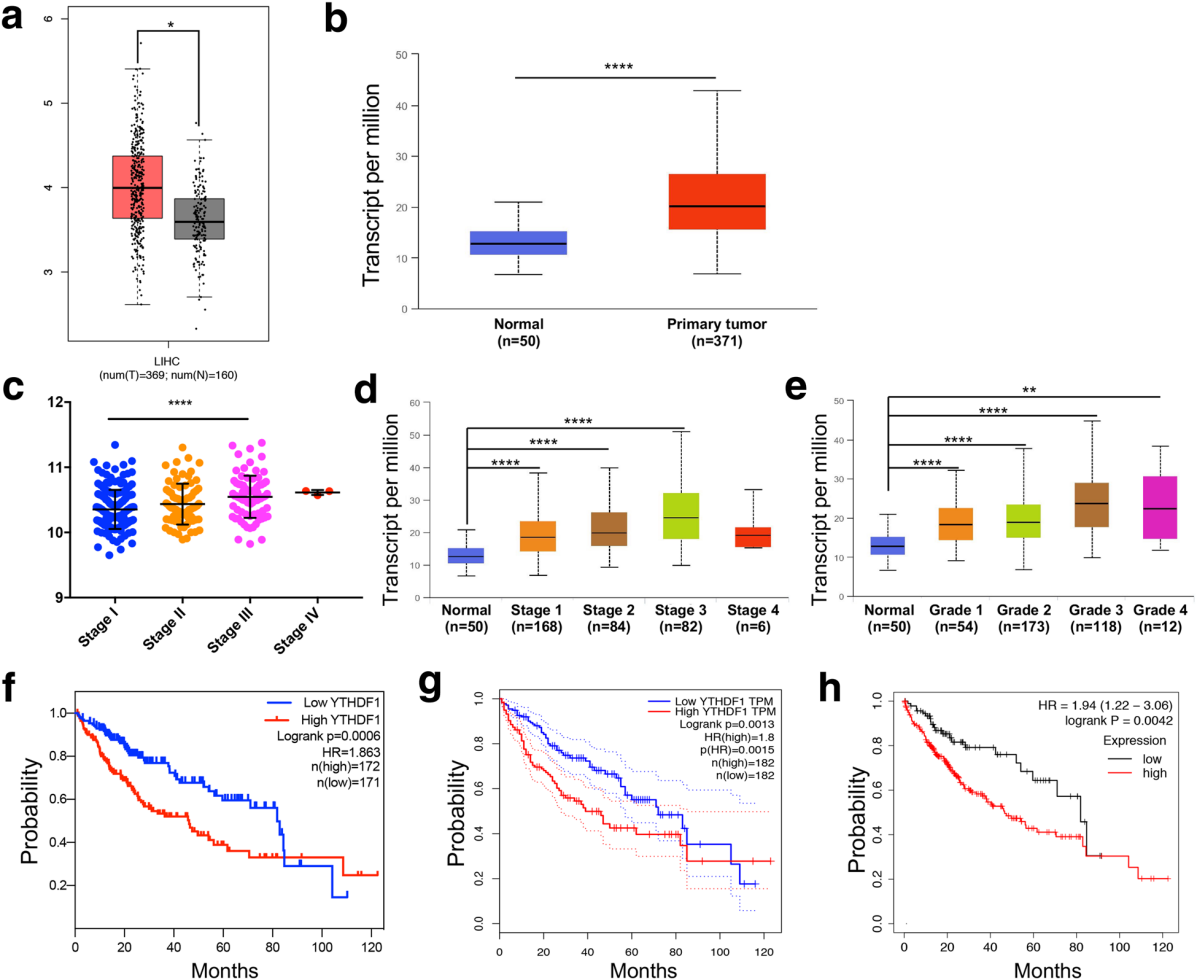
Bioinformatics analysis indicated that YTHDF1 was high-expressed in HCC and was correlated with poor survival. **A** GEPIA and **B** UALCAN two databases were used to analyze the mRNA level of YTHDF1 in HCC samples and ﻿normal liver﻿ samples. **C** LinkedOmics and **D** UALCAN two databases were used to analyze the mRNA level of YTHDF1 in different stages of HCC. **E** The mRNA level of YTHDF1 in different grades of HCC was analyzed in UALCAN database. **F** LinkedOmics, **G** GEPIA, and **H** Kaplan–Meier Plotter databases were used for survival analysis to evaluate the influence of YTHDF1 on the overall survival of HCC patients. *P < 0.05, **P < 0.01, ****P < 0.0001

To explore the expression and clinical correlation of YTHDF1 in HCC, a range of bioinformatics databases were analyzed. We found that YTHDF1 was significantly highly expressed in HCC tissue samples compared with normal liver tissues at mRNA level from GEPIA and UALCAN two databases (Fig. 1A, B). Further analysis from LinkedOmics and UALCAN databases revealed that the mRNA level of YTHDF1 was relatively high in advanced and high-grade HCC tissues, while relatively low in early and low-grade HCC tissues (Fig. 1C–E). In addition, three bioinformatics databases including LinkedOmics, GEPIA, and Kaplan–Meier Plotter were used to conduct survival analysis. The results showed that the HCC patients with high-expression of YTHDF1 had a worse overall survival than patients with low-expression (Fig. 1F–H).

**Fig. 2:**
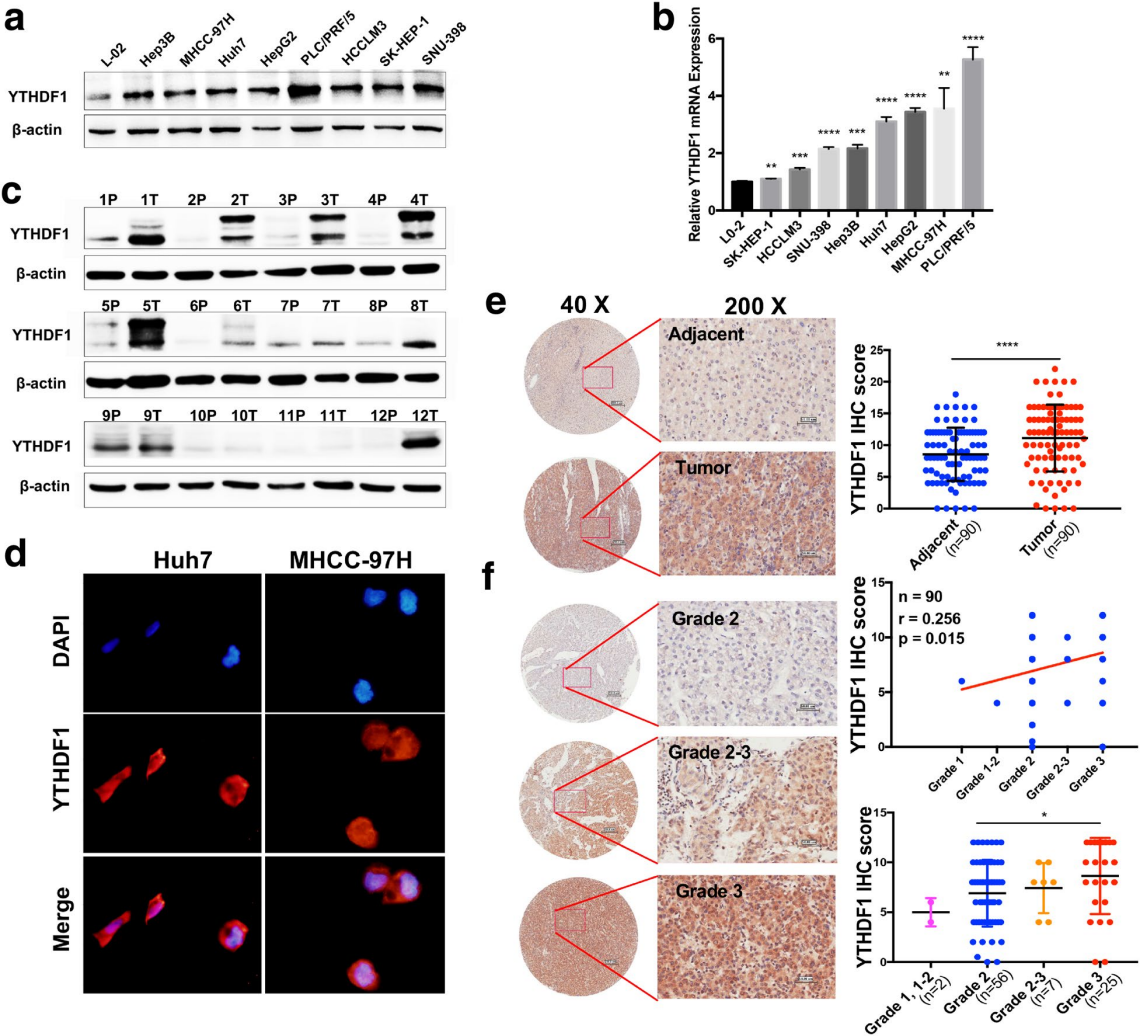
YTHDF1 was overexpressed in HCC and was associated with tumor grade. **A** The protein level of YTHDF1 detected by western blot and **B** the mRNA level of YTHDF1 detected by q-RT PCR in a normal liver cell and 8 different HCC cell lines. **C** The protein level of YTHDF1 was detected by western blot in 12 paired HCC tissues marked by T and adjacent nontumorous tissues marked by P. **D** The subcellular location of YTHDF1 was examined by immunofluorescence assay. **E** Representative immunohistochemical (IHC) staining images of YTHDF1 expression in 90 HCC tissues and paired adjacent nontumorous tissues (**F**) or in different grades of HCC tissues, conducted by tissue microarrays. Scatter plot analysis and clinicopathological correlation analysis of YTHDF1 IHC score in HCC tissues and nontumorous tissues were performed. Scale bar, 200 μm (40X) and 50 μm (200X). *P < 0.05, **P < 0.01, ***P < 0.001, ****P < 0.0001

Given the above results analyzed from bioinformatics databases, we implemented multiple assays to verify the expression and clinicopathological correlation of YTHDF1 in HCC. First, the expression of YTHDF1 in a normal liver cell and various HCC cell lines at the protein level were detected by western blot (Fig. 2A) and mRNA level were detected by q-RT PCR (Fig. 2B). The results showed that YTHDF1 was high-expressed in HCC cells at the protein level and mRNA level, including Hep3B, MHCC-97H, Huh7, HepG2, PLC/PRF/5, HCCLM3, SK-HEP-1 and SNU-398, compared with a normal liver cell L02. Simultaneously, it was found that YTHDF1 was also significantly up-regulated in 12 HCC tissues compared with nontumorous tissues **(**Fig. 2C). The results of immunofluorescence assay indicated that YTHDF1 was distributed in both cytoplasm and nucleus (Fig. 2D).

To further confirm the in situ expression and clinicopathological features of YTHDF1 in patients with HCC, we analyzed a HCC tissue microarray containing 90 tumorous tissues and adjacent nontumorous tissues. The results showed that YTHDF1 was remarkably up-regulated in HCC tissues (*P* < 0.0001), and the subcellular location of YTHDF1 was consistent with previous immunofluorescence results (Fig. 2E). Besides, the expression of YTHDF1 was correlated with HCC grade (*P* = 0.015) (Fig. 2F). Specifically, the expression of YTHDF1 in grade 3 HCC was higher than that in grade 2 HCC (Fig. 2F, low right).

Taken together, these results indicated that the expression of YTHDF1 was significantly up-regulated in HCC and was associated with tumor grade. The survival of HCC patients with YTHDF1 high-expression is poor.

### YTHDF1 silencing inhibited the tumor properties of HCC cells in vitro and in vivo

**Fig. 3:**
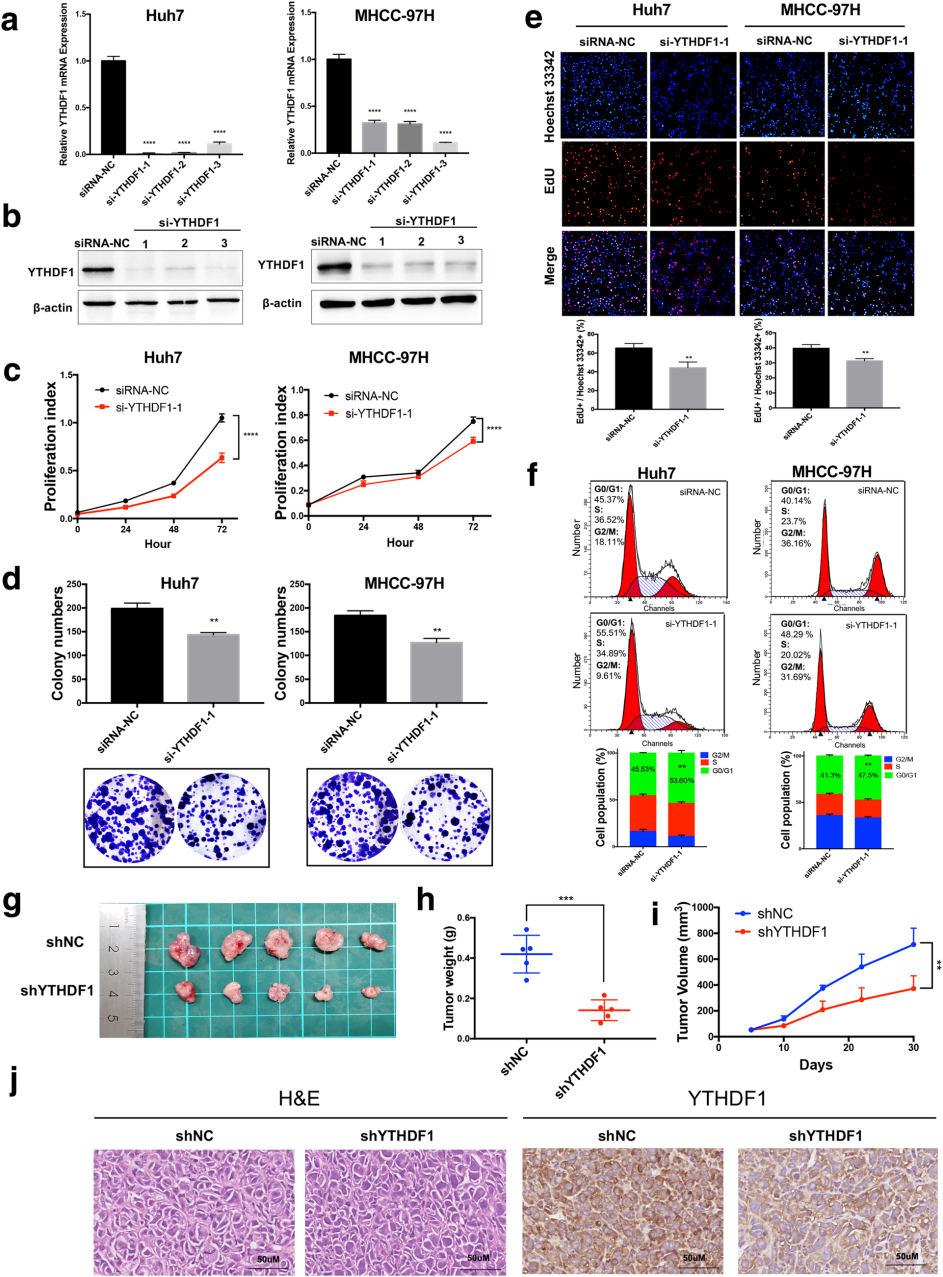
Downregulation of YTHDF1 inhibited the proliferation and cell cycle of HCC cells. **A** The mRNA level and **B** protein level of YTHDF1 were significantly inhibited by three si-YTHDF1s. **C** Cell proliferation was detected by CCK8 assay in Huh7 and MHCC-97H cells transfected with si-YTHDF1. **D** Colony-forming ability of Huh7 and MHCC-97H cells treated with si-YTHDF1 was measured by colony-forming assay. **E** DNA replication ability was assessed by EdU assay in Huh7 and MHCC-97H cells transfected with si-YTHDF1. **F** The effect of si-YTHDF1 on the cell cycle of Huh7 and MHCC-97H cells analyzed by PI staining and flow cytometry. **G** The tumors derived from Huh7 cells lacking YTHDF1 in the subcutaneous implantation mouse model, **H** and the tumor weight and **I** tumor volume were measured. **J** H&E and IHC staining of the xenograft tumors were performed. Representative images and quantitative histograms, and curve diagrams were as shown. **P < 0.01, ***P < 0.001, ****P < 0.0001

We conducted a series of assays to determine the role of YTHDF1 in HCC, especially in cell proliferation, which is essential to tumor malignant progression. In vitro, we successfully down-regulated the mRNA level and protein level of YTHDF1 in Huh7 and MHCC-97H cells via transfecting si-YTHDF1 (Fig. 3A, B). The results of CCK8 assay showed that inhibition of YTHDF1 expression significantly interfered with the proliferation of Huh7 and MHCC-97H cells (Fig. 3C). Furthermore, the colony-forming ability of HCC cells was also impaired by blocking the expression of YTHDF1 (Fig. 3D). Similarly, the results of EdU assay indicated that silencing YTHDF1 suppressed the ability of DNA replication in Huh7 and MHCC-97H cells, which was intuitively reflected by the decreased ratio of fluorescence merged cells in the YTHDF1 knockdown group compared with the control group (Fig. 3E). Besides, flow cytometry was performed to examine the effect of YTHDF1 on the HCC cell cycle process and it showed that the inhibition of YTHDF1 in Huh7 and MHCC-97H cells increased the percentage of G0/G1 cells (Fig. 3F), suggesting that the downregulation of YTHDF1 induced cell cycle arrest of HCC cells. We also constructed stable YTHDF1 knockdown cells sh-YTHDF1 using Huh7 cells. Four-week-old BALB/c male nude mice were used to perform a subcutaneous implantation experiment. The mice were injected subcutaneously with 2 × 10^6^ Huh7 cells treated with sh-YTHDF1 or sh-NC. The size of the tumor was then observed and measured every 5–8 days until the tumor grew to an appropriate size (Fig. 3G). We found that the tumor weight (Fig. 3H) and volume (Fig. 3I) in the YTHDF1 knockdown group were significantly lower than those in the control group. Furthermore, hematoxylin and eosin (H&E) staining of the subcutaneously implanted tumors showed the morphology of tumor cells, and IHC staining confirmed the downregulation of YTHDF1 in the shYTHDF1 group (Fig. 3J).

**Fig. 4:**
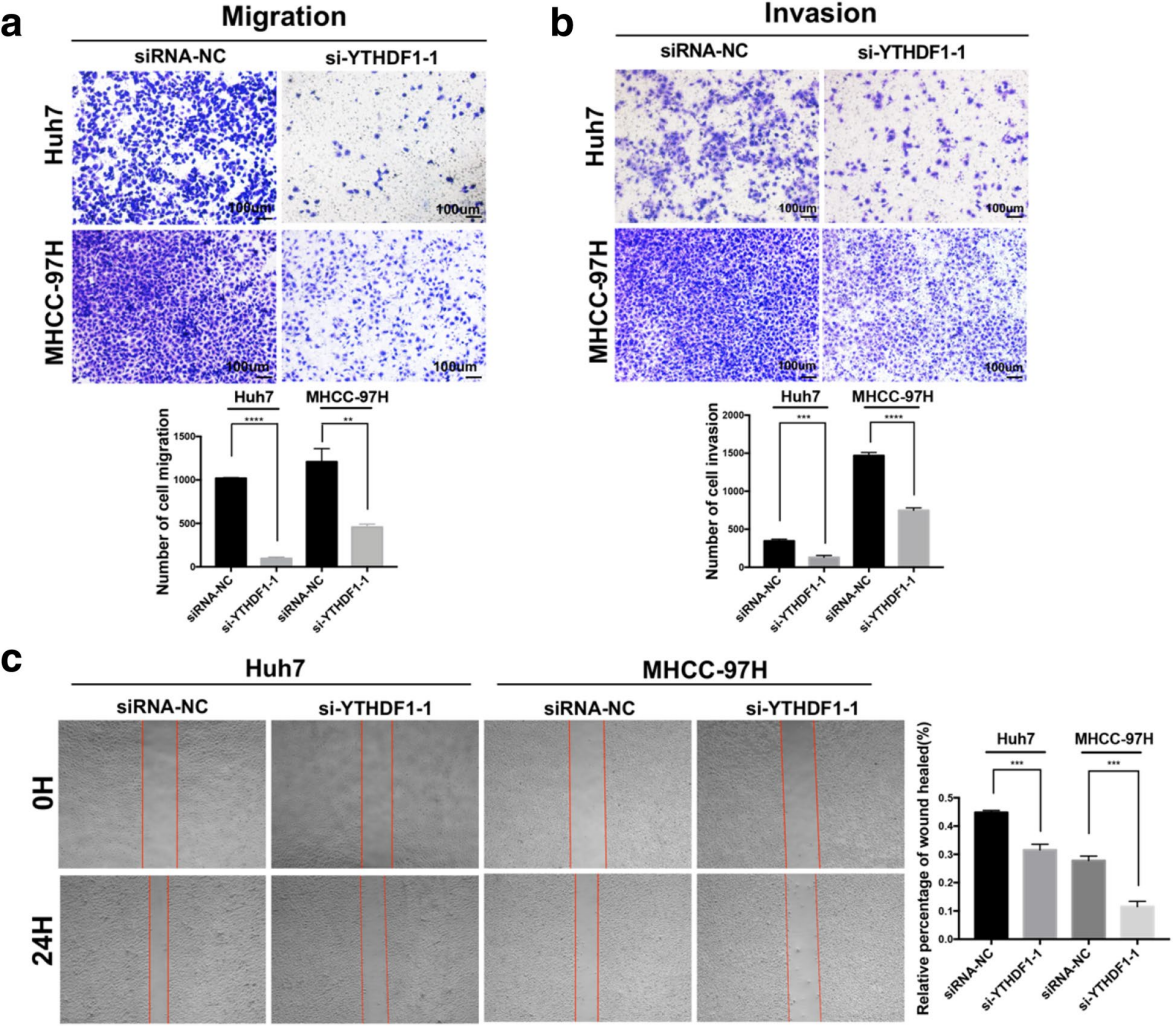
Downregulation of YTHDF1 inhibited the migration and invasion of HCC cells. **A** Cell migration ability and **B** invasion ability of Huh7 and MHCC-97H cells treated with si-YTHDF1 were evaluated by Transwell assay. **C** Wound healing assay was performed to measure the migration capacity of the cells with YTHDF1 knockdown in 24 h. Representative images and quantitative histograms were indicated. **P < 0.01, ***P < 0.001, ****P < 0.0001

Accumulated evidence shows that RNA m^6^A modification not only participates in the proliferation of cancer cells, but also affects the metastasis of multiple cancer cells [21–25]. To investigate the potential of YTHDF1 on HCC metastasis, we evaluated it in vitro. The results of transwell migration assay showed that the migratory ability of Huh7 and MHCC-97H cells were significantly suppressed in response to the downregulation of YTHDF1 (Fig. 4A). The invasive capacity was measured by transwell assay equipped with Matrigel, which contained various matrix proteins and was used to simulate cellular basement membrane in vivo. The results showed that the number of cells moving across transwell Matrigel was lessened apparently in the YTHDF1 knockdown group (Fig. 4B), indicating that the inhibition of YTHDF1 expression destroyed the invasive ability of HCC cells. Additionally, similar results were demonstrated by wound healing assay. The migratory proportion of Huh7 and MHCC-97H cells in the YTHDF1-deficient group was decreased markedly than that in the control group after cultured in serum-free medium for 24 h (Fig. 4C).

Collectively, these results suggested that YTHDF1 promoted the proliferation, cell cycle, migration and invasion of HCC cells.

### YTHDF1 facilitated the proliferation of HCC cells by activating PI3K/AKT/mTOR signaling pathway

**Fig. 5:**
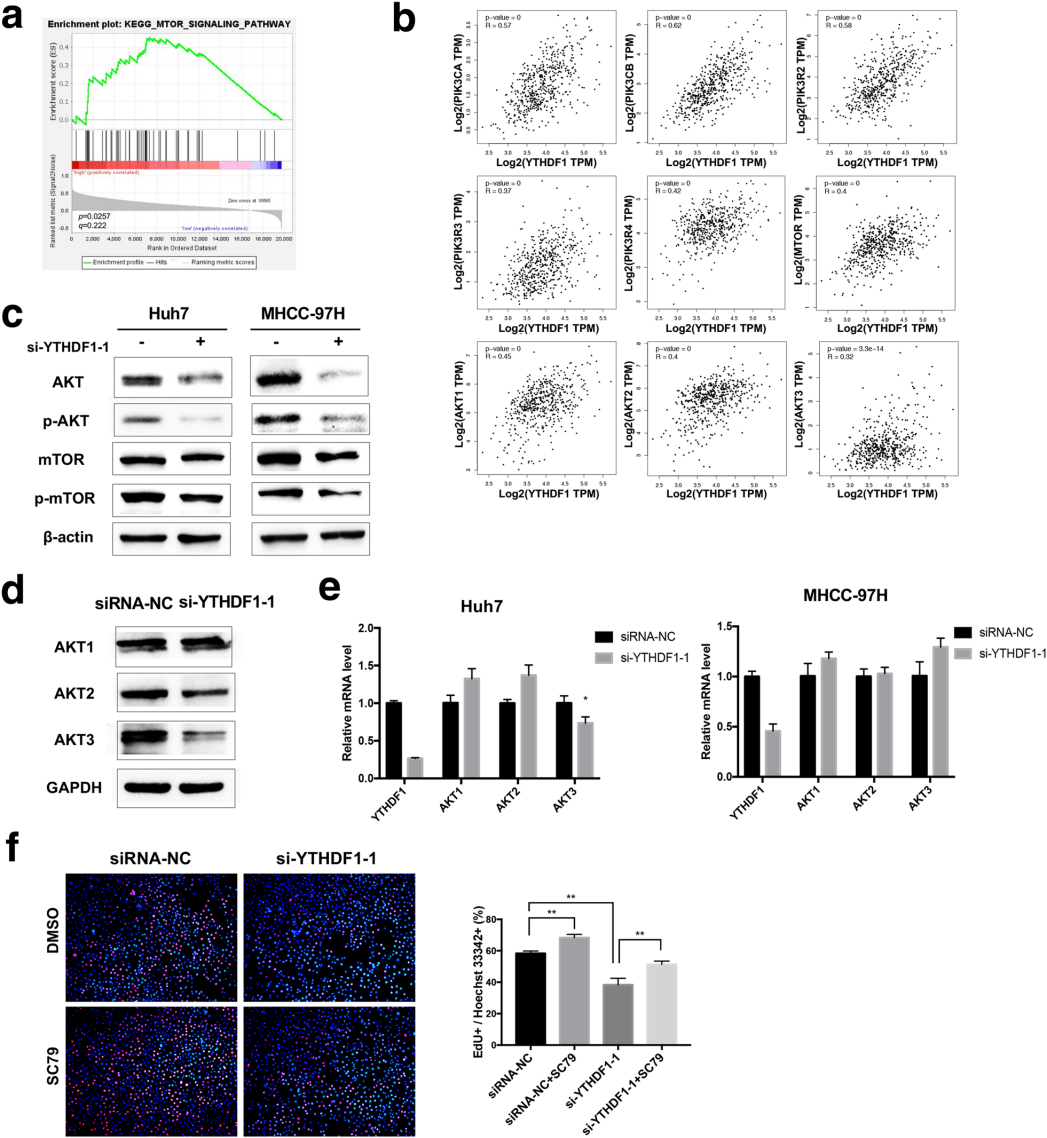
YTHDF1 facilitated the proliferation of HCC cells by activating PI3K/AKT/mTOR signaling pathway. **A** The results of GSEA indicated that PI3K/AKT/mTOR signaling pathway was enriched in HCC with YTHDF1-overexpression. **B** The correlation analysis between YTHDF1 and key molecules of PI3K/AKT/mTOR signaling pathway in HCC was performed by using GEPIA. **C** Western blot was performed to detect the protein levels of AKT, p-AKT, mTOR, p-mTOR in Huh7 and MHCC-97H cells with YTHDF1 knockdown. **D** Western blot was performed to detect the protein levels of AKT1, AKT2, and AKT3 in MHCC-97H cells with YTHDF1 silencing. **E** q-RT PCR was performed to measure the mRNA levels of AKT1, AKT2, and AKT3 in Huh7 and MHCC-97H cells with YTHDF1 silencing. **F** YTHDF1-deficient MHCC-97H cells treated with 4ug/ml SC79 for 1 h, cell proliferation was measured by Edu assay. Representative images and quantitative histograms were shown. *P < 0.05, **P < 0.01

To deeply excavate the molecular mechanism of YTHDF1 involved in the progression of HCC, an RNA-seq data set of HCC patient samples from TCGA was collected, which including 186 cases of YTHDF1 high expression and 185 cases of YTHDF1 low expression. Functional enrichment of gene pathways was performed by GSEA using the KEGG gene sets, which classified most genes through signaling pathways and biological processes. The analysis results exhibited that the PI3K/AKT/mTOR signaling pathway was significantly enriched in the YTHDF1 high expression subset (Fig. 5A). Additionally, correlation analysis by GEPIA showed that multiple key transcripts in the PI3K/AKT/mTOR signaling pathway, such as PIK3CA, PIK3CB, PIK3R2, PIK3R3, PIK3R4, mTOR, AKT1, AKT2, and AKT3, were positively correlated with YTHDF1 transcripts in HCC (Fig. 5B). To confirmed the result of this bioinformatic analysis, subsequent western blot assay was performed and the results indicated that downregulation of YTHDF1 apparently inhibited the protein levels of AKT, p-AKT, mTOR, and p-mTOR (Fig. 5C). Then, we tested the effect of YTHDF1 on the expression of different AKT isoforms. The results of western blot assay showed that the protein levels of AKT2 and AKT3 were inhibited by YTHDF1 silencing, while AKT1 did not change significantly (Fig. 5D). However, q-RT PCR results showed that only the mRNA level of AKT3 in huh7 cells with YTHDF1 knockdown was moderately down-regulated, the mRNA levels of AKT1, AKT2, and AKT3 in MHCC-97H cells with YTHDF1 knockdown did not change or even upregulated (Fig. 5E). Therefore, the downregulation of YTHDF1 only inhibited the translation of AKT2 and AKT3, but did not involve the transcriptional level. Furthermore, the results of EdU assay showed that SC79, one of AKT activators, could partially restore the suppressed effect on cell proliferation induced by YTHDF1 inhibition (Fig. 5F). These results demonstrated that YTHDF1 promoted the proliferation of HCC cells by regulating the activation of PI3K/AKT/mTOR signaling pathway.

### YTHDF1 promoted the migration and invasion ability of HCC cells via inducing EMT

**Fig. 6:**
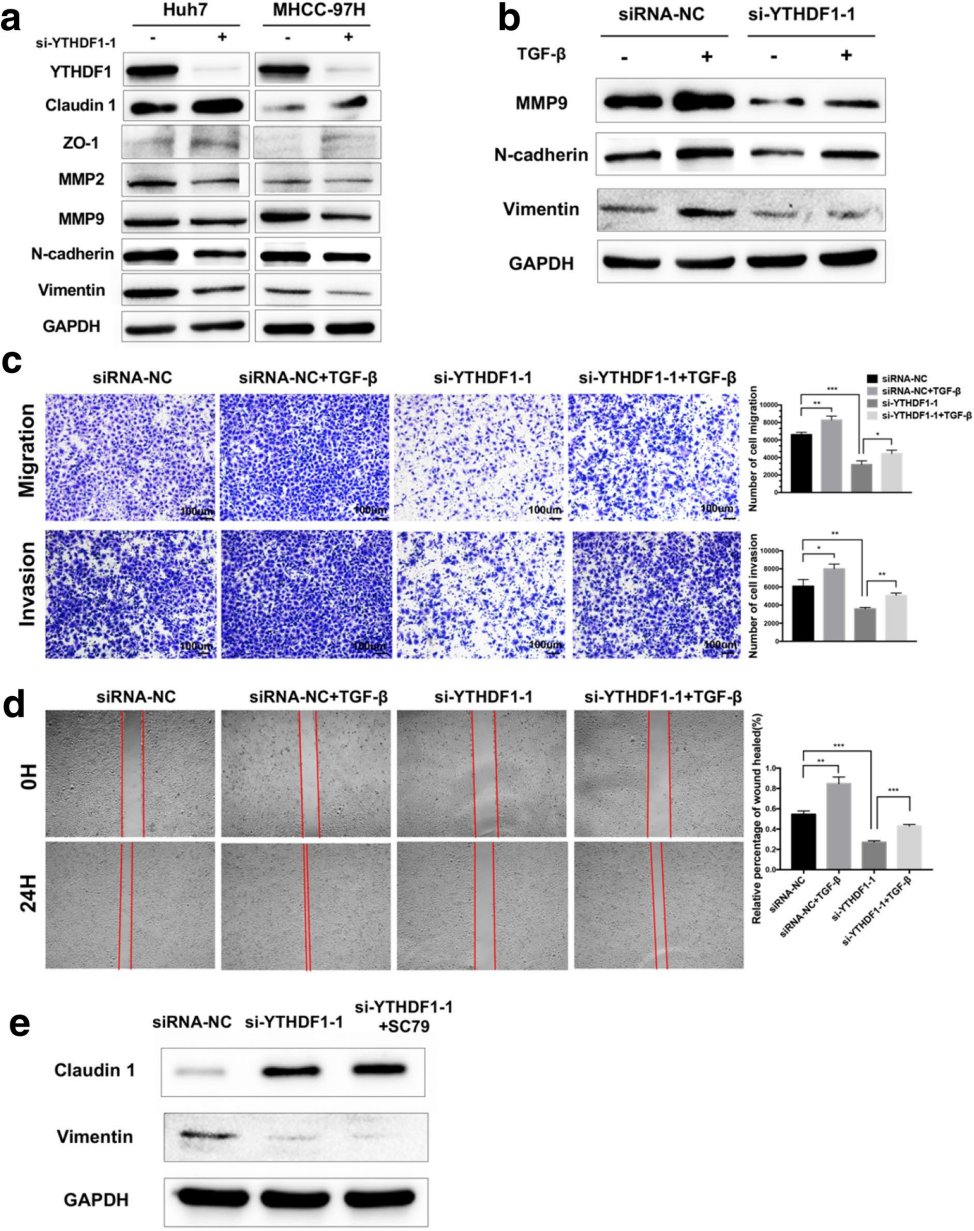
YTHDF1 promoted the migration and invasion of HCC cells via inducing EMT. **A** Western blot was performed to detect the influence of si-YTHDF1 on protein levels of EMT markers in Huh7 and MHCC-97H cells. **B** The MHCC-97H cells with YTHDF1 knockdown were treated with 10 ng/ml TGF-β for 48 h, then measured the protein levels of EMT markers by western blot. **C** Transwell assay and **D** wound healing assay were performed to assess the migration and invasion ability of MHCC-97H cells with YTHDF1 silencing that were pretreated with 10 ng/ml TGF-β for 48 h. **E** Western blot was performed to detect the protein levels of Claudin 1 and Vimentin in MHCC-97H cells treated with or without 4ug/ml SC79 for 1 h upon YTHDF1 silencing. Representative images and quantitative histograms were shown. *P < 0.05, **P < 0.01, ***P < 0.001

The RNA m^6^A modification has been verified to drive the migration and invasion of cancer cells by inducing EMT [23, 26]. Considering the crucial role of EMT in tumor metastasis, it was worth to investigate whether YTHDF1 was involved in the regulation of the EMT process of HCC cells. Firstly, the expression levels of EMT-transcription factors (EMT-TFs) in Huh7 and MHCC-97H cells with YTHDF1 silencing were detected. The results showed that the expression levels of epithelial cell markers Claudin 1 and zonula occludens protein 1 (ZO-1) were significantly up-regulated, while the expression levels of mesenchymal cell markers matrix metallopeptidase 2 (MMP2), matrix metallopeptidase 9 (MMP9), N-cadherin, and Vimentin were suppressed after YTHDF1 knockdown (Fig. 6A). It suggested that YTHDF1 could induce EMT in HCC cells. To ascertain whether EMT mediates the role of YTHDF1 in the migration and invasion of HCC cells, we treated MHCC-97H cells with TGF-β to induce EMT. The results of western blot assay indicated that TGF-β successfully induced the EMT, and the knockdown of YTHDF1 attenuated the EMT induced by TGF-β (Fig. 6B). Furthermore, the results of transwell assay and wound healing assay showed that TGF-β treatment markedly increased the number of migratory and invasive cells and partly counteracted the inhibitory effect of YTHDF1-deficient on the migration and invasion capabilities of MHCC-97H cells (Fig. 6C, D). Previous studies found that the AKT/mTOR signaling pathway enables inducing the EMT process [27]. To explore whether AKT mediates the induction of EMT by YTHDF1 in HCC, a rescue experiment was performed. The results of western blot assay showed that MHCC-97H cells treated with SC79 to overexpress AKT could not rescue the expression changes of Claudin 1 and Vimentin upon YTHDF1 knockdown (Fig. 6E). In short, EMT was partly responsible for the promotion effect of YTHDF1 on the migration and invasion of HCC cells, and this regulation of EMT by YTHDF1 does not depend on the AKT/ mTOR signaling pathway.

## Discussion

The study of epigenetic modification, especially RNA m^6^A modification in tumors, has been developed rapidly in recent years. YTHDF1, as a pivotal “reader” protein in m^6^A regulators, plays important role in different kinds of cancers [11–16]. However, the exact and thorough role and molecular mechanism of YTHDF1 on the occurrence and development of HCC is still not fully understood.

In this study, we uncovered that YTHDF1 was significantly overexpressed in HCC cells and tissues and was related to the grading of HCC by using databases analysis, western blot, q-RT PCR and tissue microarrays. Similarly, multiple studies have shown that YTHDF1 was significantly up-regulated in HCC and was associated with poor survival of patients with HCC by using bioinformatics and tissue samples analysis [28–35]. Previous studies demonstrated that YTHDF1 facilitated the proliferation, migration, and invasion of MHCC-LM3 and HepG2 two HCC cells [16, 36]. Our study confirmed this promotion effect of YTHDF1 in the other two HCC cell lines Huh7 and MHCC-97H, which to a certain extent ruled out the cell dependence of YTHDF1 on this carcinogenic role of HCC. Interestingly, our study also demonstrated that YTHDF1 facilitated HCC cell cycle progression, which was predicted and indicated by previous studies [28, 29, 36].

The PI3K/AKT/mTOR signaling pathway plays a broad spectrum role in physiology, and its dysregulation often leads to multiple diseases, including cancer [37–40]. There is evidence that the PI3K/AKT/mTOR pathway mediates the function of m^6^A “writers” METTL3 and METTL14 in the progression of gastric cancer and retinoblastoma [41, 42]. In HCC, the PI3K/AKT/mTOR pathway is overexpressed in 40–50% of samples [43]. The abnormal activation of this well-characterized pathway affects cell proliferation and metabolism, and leads to less tumor differentiation, poor prognosis, and earlier recurrence in HCC, but has nothing to do with the underlying etiology of HCC [43]. Based on the previous study [36], we found that the PI3K/AKT/mTOR signaling pathway might be associated with the carcinogenic role of YTHDF1 in HCC by using bioinformatic GSEA. Then, the results of western blot assay indicated that the downregulation of YTHDF1 significantly decreased the expression of key pathway proteins AKT, p-AKT, mTOR, and p-mTOR. Notably, we found that YTHDF1 mainly up-regulated the translation of AKT2 and AKT3, rather than their transcription. Indeed, a previous study identified that YTHDF1 was a translation initiation promotor [10]. Therefore, we speculated that YTHDF1 might directly interact with AKT2 and AKT3 to promote the progression of HCC. Additionally, treatment with a specific AKT activator SC79 partly rescued the suppression of HCC cell proliferation in YTHDF1-deficient cells. Our study also found that high-expressed YTHDF1 samples from TCGA were significantly enriched in the WNT/β-catenin signaling pathway in the BIOCARTA database (Additional file 1: Figure S1A). The results of western blot assay indicated that the downregulation of YTHDF1 strikingly suppressed the expression of key downstream proteins of WNT/β-catenin pathway such as Cyclin D1 and CD44 in HCC (Additional file 1: Figure S1B). This result is consistent with previous study [16]. It is suggested that there may be multiple signaling pathways and molecular mechanisms involved in the carcinogenesis of HCC induced by YTHDF1, which needs to be further deep explored.

EMT is a reversible cellular process that is activated in almost all malignant tumor progression [44]. Universally, it is believed that EMT plays an important role in tumor metastasis due to the enhanced ability of cell dissemination and motion [44, 45]. Studies showed that m^6^A modification was involved in the EMT process in various cancers, including gastric cancer, lung cancer, nasopharyngeal carcinoma, and colorectal cancer [23, 46–48]. Here, we found that the suppression of YTHDF1 could up-regulate the expression of epithelial cell markers Claudin 1 and ZO-1 and inhibit the expression of mesenchymal cell markers MMP2, MMP9, N-cadherin, and Vimentin in HCC cells. Furthermore, the knockdown of YTHDF1 attenuated the EMT induced by TGF-β. Previously, Lin et al. [26] revealed that downregulation of m^6^A by inhibiting the expression of METTL3 disturbed the EMT process. Notably, they also found that Snail, a key EMT-TFs, can be combined by YTHDF1 to its mRNA CDS, thereby promoting its translation in HeLa and HepG2 cells [26]. It is suggested that YTHDF1 may induce EMT activation in HCC. Similarly, Bian et al. [36] also preliminarily explored the regulatory relationship between YTHDF1 and EMT in HCC. Considering that there was evidence that the AKT/mTOR signaling pathway promoted the EMT process [27], we detected whether YTHDF1 induced EMT by activating AKT. The results showed that HCC cells treated with the AKT inducer SC79 could not rescue the changes in the protein abundance of Claudin 1 and Vimentin after YTHDF1 knockdown. Therefore, our study further confirmed that YTHDF1 induced the EMT process in an AKT-independent manner to promote the migration and invasion of HCC cells. However, whether YTHDF1 directly interacts with key molecules in an m^6^A-dependent manner to regulate PI3K/AKT/mTOR signaling pathway and EMT remains to be further elucidated.

## Conclusions

In summary, our study demonstrates that YTHDF1 is significantly up-regulated in HCC and is correlated with HCC grade. It may be a potential prognostic biomarker and therapeutic target of HCC. YTHDF1 facilitates the proliferation of HCC cells by activating PI3K/AKT/mTOR signaling pathway and promotes the migration and invasion of HCC cells by inducing EMT.

## Supplementary Information

**Additional file 1: Figure S1.** YTHDF1 regulates WNT/β-catenin signaling pathway in HCC. (A) GSEA predicted that WNT pathway was enriched in HCC with YTHDF1 high-expression. (B) Western blot showed that YTHDF1 inhibition decreased the protein expression of WNT pathway downstream molecules Cyclin D1 and CD44 in Huh7 and MHCC-97H cells.

## Data Availability

The datasets used and/or analyzed during the current study are available from the corresponding author on reasonable request.

## Abbreviations

HCC: Hepatocellular carcinoma
m^6^A: N6-methyladenosine
YTHDF1: YTH N6-methyladenosine RNA binding protein 1
PI3K: Phosphoinositide 3‐kinase
AKT: AKT serine/threonine kinase
mTOR: Mammalian target of rapamycin
EMT: Epithelial-mesenchymal transition
METTL3/14: Methyltransferase like 3 and 14
WTAP: WT1 associated protein
FTO: FTO alpha-ketoglutarate dependent dioxygenase
ALKBH5: AlkB homolog 5, RNA demethylase
PD-L1: Programmed cell death 1 ligand 1
EMT-TFs: EMT-transcription factors
TGF-β: Transforming growth factor-beta
PI: Propidium iodide
TCGA: The cancer genome atlas
GSEA: Gene set enrichment analysis
KEGG: Kyoto encyclopedia of genes and genomes
H&E: Hematoxylin and eosin
IHC: Immunohistochemical
MMP2: Matrix metallopeptidase 2
MMP9: Matrix metallopeptidase 9
ZO-1: Zonula occludens protein 1
