# Supplementary material for: YTHDF1 promotes hepatocellular carcinoma progression via activating PI3K/AKT/mTOR signaling pathway and inducing epithelial-mesenchymal transition
Source: Exp Hematol Oncol. 2021 Jun 4;10:35. doi: 10.1186/s40164-021-00227-0 (PMC8176587; doi:10.1186/s40164-021-00227-0)
Supplement: Supplementary file 1 — Additional file 1: Figure S1. YTHDF1 regulates WNT/β-catenin signaling pathway in HCC. (A) GSEA predicted that WNT pathway was enriched in HCC with YTHDF1 high-expression. (B) Western blot showed that YTHDF1 inhibition decreased the protein expression of WNT pathway downstream molecules Cyclin D1 and CD44 in Huh7 and MHCC-97H cells. [file 40164_2021_227_MOESM1_ESM.docx]

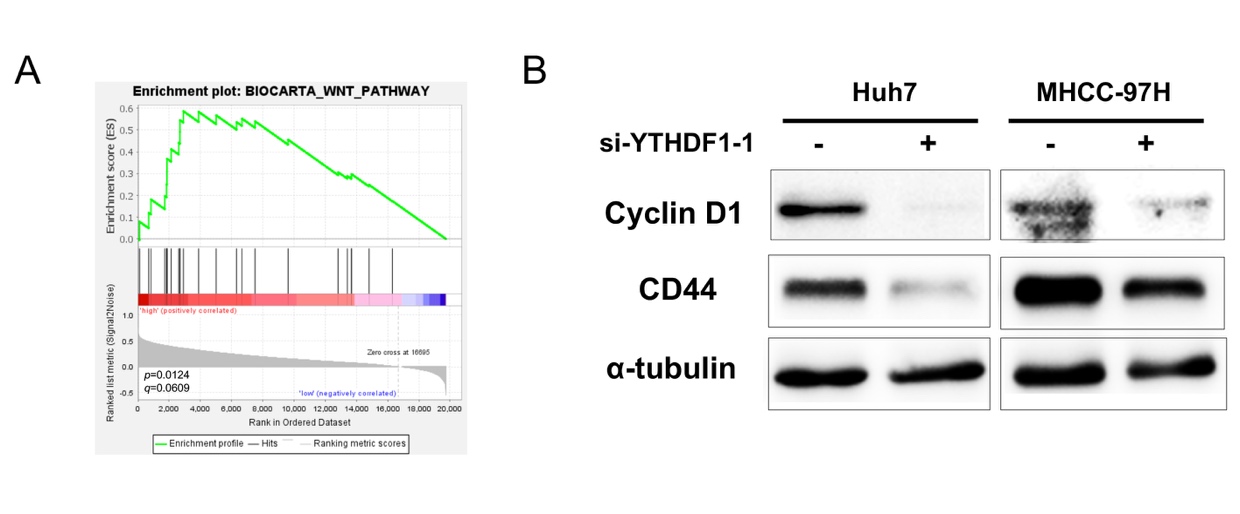


**Supplementary Figure S1: YTHDF1 regulates WNT/β-catenin signaling pathway in HCC.** (A) GSEA predicted that WNT pathway was enriched in HCC with YTHDF1 high-expression. (B) Western blot showed that YTHDF1 inhibition decreased the protein expression of WNT pathway downstream molecules Cyclin D1 and CD44 in Huh7 and MHCC-97H cells.
